# A Treatise on the Diseases of the Heart and Great Vessels, and on the Affections Which May Be Mistaken for Them

**Published:** 1842-10-15

**Authors:** 


### BIBLIOGRAPHICAL NOTICES.

*A Treatise on the Diseases of the Heart and Great Vessels, and on the Affections which may be mistaken for them.* By J. Hope, M. D., F. R. S. First American, from the third London Edition. With Notes and a Detail of Recent Experiments. By C. W. Pennock, M. D., Attending Physician to the Philadelphia Hospital, Blockley. Philadelphia: Haswell & Johnson, 1842. 1 vol. 8vo., pp. 572.

The excellent work of Dr. Hope is introduced to the profession of this country, by Dr. Pennock, who has incorporated with it a very considerable amount of valuable additional matter. Dr. Hope's work has been long recognised as the most complete and elaborate treatise on the subject, and we have no hesitation in saying that in importance and value it is not surpassed by any book on medicine lately offered to the profession. The editorial additions of Dr. Pennock are beyond the ordinary labours of 'American editors.' His distinguished researches as an experimentalist, and his long and assiduous devotion to the pathology of diseases of the heart, rendered him peculiarly qualified for this task, and he has discharged it most ably and judiciously. Dr. Pennock's additions contain a large amount of original matter, and, besides, extracts from the writings of other pathologists, as C. J. B. Williams, Bizot, and others. Among the original matter of the editor, is the report of the Experiments on the Action of the Heart, performed by him, which were first published in this Journal, Vol. 2d, No. 44, and are there commented on at length. A description of a previously undescribed form of dissecting aneurism is also given, illustrated by several cases and a plate. The running commentaries by Dr. Pennock on the text of Dr. Hope, supply some important deficiencies in the author, and present with entire completeness, the actual state of knowledge respecting the pathology of diseases of the heart.

